# Supplementary figures and images for: The complex hybrid origins of the root knot nematodes revealed through comparative genomics
Source: PeerJ. 2014 May 6;2:e356. doi: 10.7717/peerj.356 (PMC4017819; doi:10.7717/peerj.356)

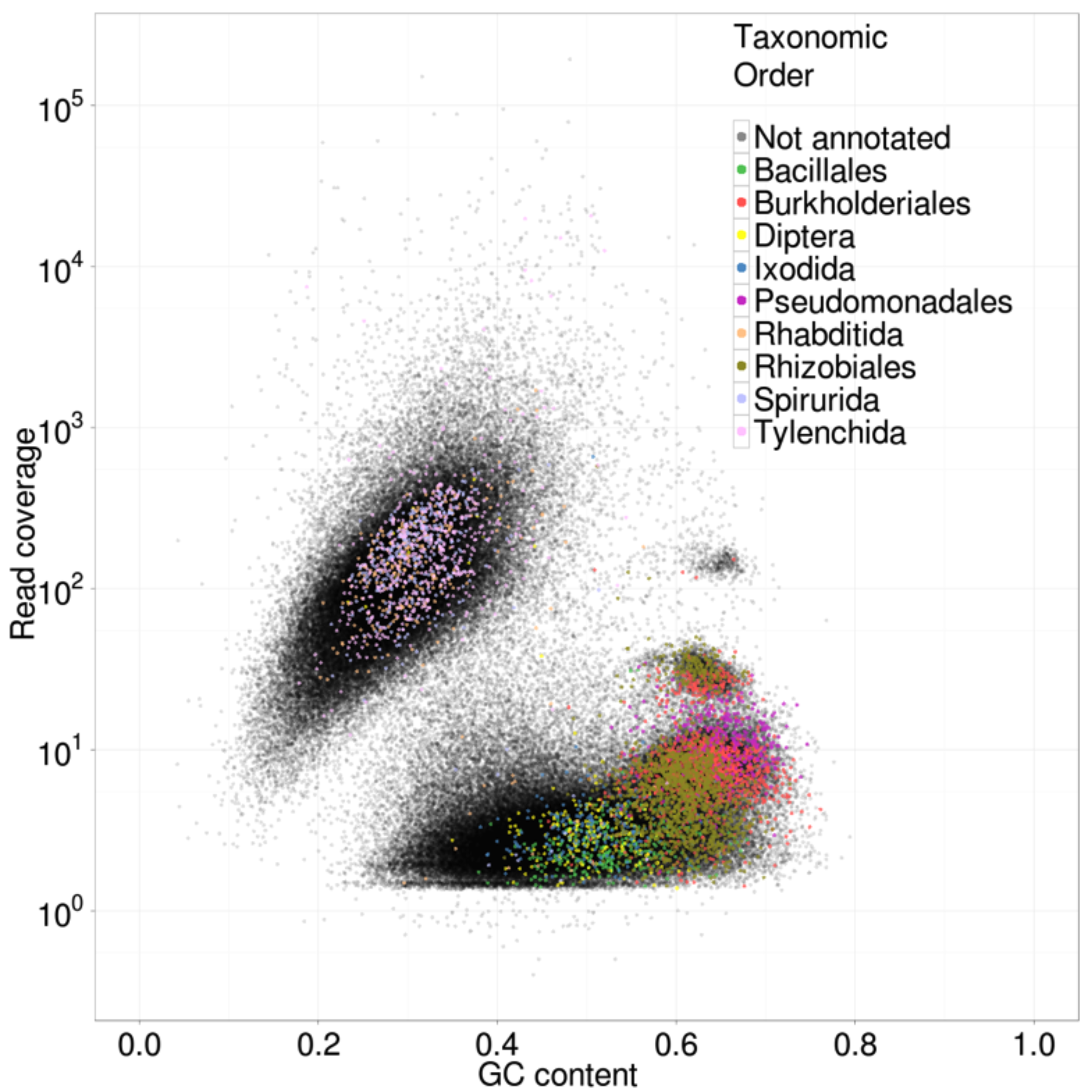

Supplement: Figure S1 — The contigs from primary assembly of the raw data from Meloidogyne floridensis were annotated with their GC% and their read coverage (estimated by mapping back all the reads used to the contig set). Ten thousand contigs >1000 bp were chosen randomly and compared, using BLASTN, to the GenBank nt nucleotide database. Any contig with a match in the database was further annotated with the taxonomic assignment (at phylum level) of the matched sequence. The contig GC% and coverage values were used to create a scatter plot in R, and contigs with taxonomic assignments were coloured according to the phylum of the best match. This plot was used to devise a data cleaning strategy prior to rigorous assembly. [file peerj-02-356-s001.png]

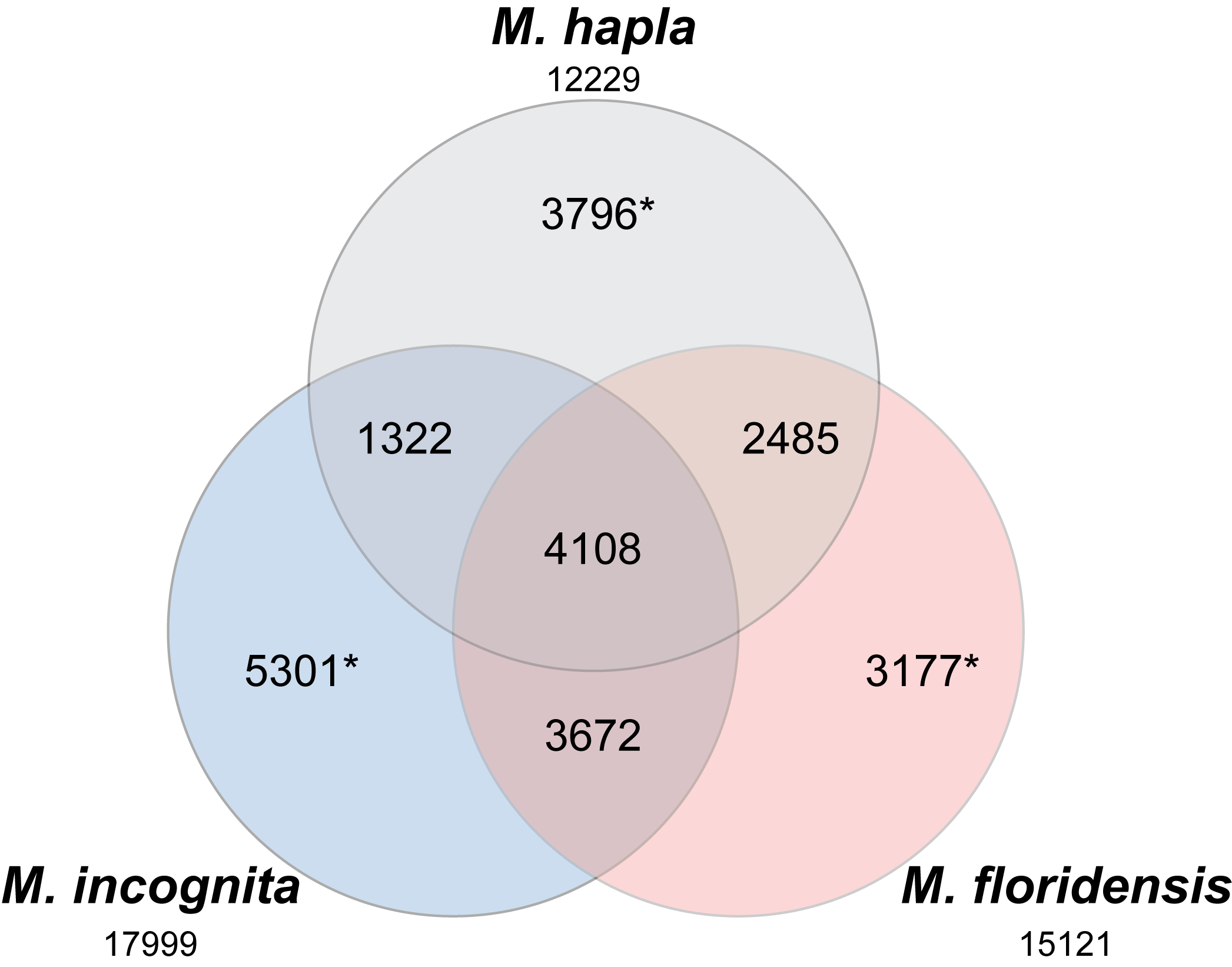

Supplement: Figure S2 — The complete proteomes of the three Meloidogyne species were clustered using InParanoid. This Venn diagram shows the numbers of clusters that had multiple species membership, and the numbers of proteins that were unique to each species (numbers marked with *). The total number of proteins input for each species are given under the species’ name. [file peerj-02-356-s002.png]
